# A Magnificent Hospital

**Published:** 1863-08

**Authors:** 


A Magnificent Hospital.—We learn that the Government has purchased one hundred and twenty acres of land a short distance north of Jeffersonville, Indiana, on the Ohio river, for the purpose of erecting an extensive hospital for our sick and wounded soldiers and sailors in the west. The various buildings (and we presume a burying ground) are to cover sixty acres, while the other sixty acres are to be laid out into a garden, to raise fruits and vegetables for the occupants of the hospital, and on which the convalescents will be employed. The hospital is intended to accommodate 800 persons; is to be made a permanent institution, and, consequently, will be constructed with the greatest care as regards convenience. It is to be furnished with gas, water from the river, and every modern improvement that experience has demonstrated of value, or the laws of hygeia can suggest as desirable. Contrabands are daily expected to arrive from the other side of the Ohio river to commence leveling the ground and preparing it for the erection of the buildings. These contrabands are to be used for the double purpose of giving them employment, and because such labor is very scarce. The situation is a commanding one, capable of being made very beautiful by the appliances of art, and is out of the reach of overflow from the river, as it is elevated land.—*Indianapolis Gazette.*

